# Histamine H4 Receptor Agonist, 4-Methylhistamine, Aggravates Disease Progression and Promotes Pro-Inflammatory Signaling in B Cells in an Experimental Autoimmune Encephalomyelitis Mouse Model

**DOI:** 10.3390/ijms241612991

**Published:** 2023-08-20

**Authors:** Abdulaziz M. S. Alsaad, Mushtaq A. Ansari, Ahmed Nadeem, Sabry M. Attia, Saleh A. Bakheet, Hatun A. Alomar, Sheikh F. Ahmad

**Affiliations:** Department of Pharmacology and Toxicology, College of Pharmacy, King Saud University, Riyadh 11451, Saudi Arabia

**Keywords:** histamine H4 receptor agonist, EAE, multiple sclerosis, inflammatory mediators, flow cytometry

## Abstract

We sought to assess the impact of 4-Methylhistamine (4-MeH), a specific agonist targeting the Histamine H4 Receptor (H4R), on the progression of experimental autoimmune encephalomyelitis (EAE) and gain insight into the underlying mechanism. EAE is a chronic autoimmune, inflammatory, and neurodegenerative disease of the central nervous system (CNS) characterized by demyelination, axonal damage, and neurodegeneration. Over the past decade, pharmacological research into the H4R has gained significance in immune and inflammatory disorders. For this study, Swiss Jim Lambert EAE mice were treated with 4-MeH (30 mg/kg/day) via intraperitoneal administration from days 14 to 42, and the control group was treated with a vehicle. Subsequently, we evaluated the clinical scores. In addition, flow cytometry was employed to estimate the impact of 4-Methylhistamine (4-MeH) on NF-κB p65, GM-CSF, MCP-1, IL-6, and TNF-α within CD19^+^ and CXCR5^+^ spleen B cells. Additionally, we investigated the effect of 4-MeH on the mRNA expression levels of Nf-κB p65, Gmcsf, Mcp1, Il6, and Tnfα in the brain of mice using RT-PCR. Notably, the clinical scores of EAE mice treated with 4-MeH showed a significant increase compared with those treated with the vehicle. The percentage of cells expressing CD19^+^NF-κB p65^+^, CXCR5^+^NF-κB p65^+^, CD19^+^GM-CSF^+^, CXCR5^+^GM-CSF^+^, CD19^+^MCP-1^+^, CXCR5^+^MCP-1^+^, CD19^+^IL-6^+^, CXCR5^+^IL-6^+^, CD19^+^TNF-α^+^, and CXCR5^+^TNF-α^+^ exhibited was more pronounced in 4-MeH-treated EAE mice when compared to vehicle-treated EAE mice. Moreover, the administration of 4-MeH led to increased expression of NfκB p65, Gmcsf, Mcp1, Il6, and Tnfα mRNA in the brains of EAE mice. This means that the H4R agonist promotes pro-inflammatory mediators aggravating EAE symptoms. Our results indicate the harmful role of H4R agonists in the pathogenesis of MS in an EAE mouse model.

## 1. Introduction

Multiple sclerosis (MS) is a disorder of the central nervous system (CNS) caused by autoimmune processes, resulting in neurological disabilities [1,2]. Patients with MS exhibit distinct lesions in CNS tissues, inflammatory infiltrates, demyelination, astrogliosis, and early axonal damage [3,4]. The pathological features of MS include inflammation, increased blood–brain barrier permeability, gliosis, multifocal demyelination plaques, and loss of axons [5]. Furthermore, the development of neuroinflammation in MS has been associated with various factors derived from antigen-presenting cells [6]. Experimental autoimmune encephalomyelitis (EAE) is an animal model commonly used to study MS. EAE mimics MS in several clinical, pathological, and immunological characteristics, such as inflammation, oligodendrocyte loss, demyelination, axonal loss, and neuronal loss [7,8,9]. Several inflammatory mediators that induce CNS activation and infiltration are involved in the etiology of EAE [10]. One of the reasons that EAE is frequently utilized as an autoimmune model of MS is because it resembles human MS and provides evidence for inflammation’s direct pathogenic role [11]. Several studies have documented its efficiency in unraveling the molecular mechanisms underlying MS and assisting in developing potential therapeutic targets against the disease in humans [12]. Multiple research studies have demonstrated immune dysfunction in both the peripheral and cerebral regions of individuals with MS and mouse models. It is widely recognized that neuroinflammatory conditions, including MS, activate immune cells in the peripheral system. These immune cells can influence neural circuitry by modulating cellular reactions in oligodendrocytes, microglia, and astrocytes through the secretion of inflammatory substances like oxidants and cytokines at the blood–brain barrier. There are reports of peripheral immune cells infiltrating brain tissue, leading to compromised blood–brain barrier permeability.

Dysfunction of cytokines has been detected in both multiple sclerosis (MS) and animal models of experimental autoimmune encephalomyelitis (EAE) [13]. Overexpression of pro-inflammatory cytokines was found in blood, cerebrospinal fluid, and central nervous system lesions of samples with MS [14]. The transcription factor NF-κB is crucial in regulating inflammation, cell viability, and inflammatory responses. Recent research has indicated that NF-κB governs the viability of resident cells within inflammatory lesions [15,16]. NF-κB triggers the expression of various inflammatory cytokines and activates multiple cell types in MS and EAE [17]. Activation of NF-κB has been demonstrated in numerous cell types in both MS and EAE [15,18]. Furthermore, studies have highlighted the significant involvement of GM-CSF, a pro-inflammatory cytokine produced by immune and tissue-resident cells [19,20] in autoimmune diseases such as MS [21,22]. It has been revealed that GM-CSF is highly abundant in the cerebrospinal fluid of individuals with MS [23].

Multiple studies have documented the presence of MCP-1 expression in the central nervous system (CNS) of individuals with multiple sclerosis (MS) [24,25]. MCP-1 is associated with the manifestation of acute disease symptoms and initiating blood–brain barrier breakdown [26]. Furthermore, MCP-1 expression precedes the infiltration of monocytes into the CNS and the onset of clinical signs [27]. TNF-α, a pleiotropic cytokine, plays a role in stimulating the proliferation of oligodendrocyte progenitors and remyelination [28]. It is actively upregulated in the serum and active lesions of MS patients during the attack stage of experimental autoimmune encephalomyelitis (EAE) [29]. IL-6, a crucial cytokine in the induction phase of EAE pathogenesis [30], has been reported to promote clinical signs of EAE [31].

The Histamine H4 Receptor (H4R) belongs to the G protein-coupled receptor subfamily of histamine receptors and represents the most recent discovery in this group [32,33,34]. In the past decade, there has been a growing interest in pharmacological research focused on H4R for treating immune and inflammatory disorders [35]. H4R is expressed in immune cells and various tissues and plays a functional role in inflammatory diseases and immuno-modulatory pathways [36,37]. Recent investigations have elucidated the involvement of the newly discovered H4R in the inflammatory effects of histamine, especially in immune cells where it is predominantly expressed [38,39]. H4R exhibits high expression levels in the bone marrow, spleen, and other tissues, and numerous studies have supported its potential as an H4R agonist [40,41]. H4R is functionally present in neurons within the central nervous system (CNS) [42]. Moreover, it has been reported that H4R is primarily expressed in the cerebellum and, to a lesser extent, in the hippocampus [43].

Among specific agonists, 4-methylhistamine (4-MeH) is the most widely used selective H4R agonist [44]. Recently, stimulation 4-MeH was found to surge the production of pro-inflammatory cytokines [45]. Moreover, 4-MeH upregulated the expression of pro-inflammatory cytokines and promoted the expression of co-stimulatory CD80/CD86 molecules and CD28 signaling [46,47]. However, to our knowledge, no studies have investigated the role of H4R agonists in mice with EAE yet. Therefore, in the present study, we aimed to assess the impact of 4-MeH on experimental autoimmune encephalomyelitis (EAE) using a mouse model and investigate the underlying mechanisms involved.

## 2. Results

### 2.1. 4-MeH Aggravates EAE Severity in EAE Mice

**Figure 1:**
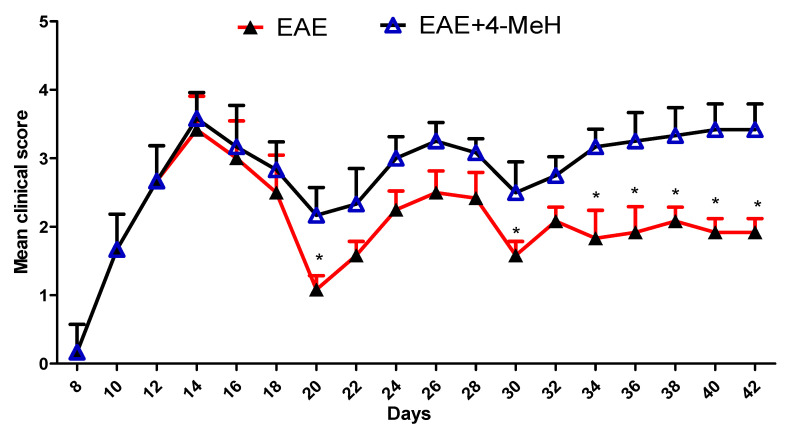
The effect of 4-MeH on the clinical score in EAE mice. SJL mice were immunized with PLP139-151 to induce EAE. Then, the mice were intraperitoneally treated with 4-MeH (30 mg/kg) or vehicle daily from day 14 to day 42. All data are shown as mean ± SD (n = 6). The level of significance was set at * *p* < 0.05.

The impact of the H4R agonist on EAE was examined in our study. Figure 1 illustrates the clinical scores of EAE mice treated with either the vehicle or 4-MeH. The findings demonstrated a significant increase in the clinical scores of EAE mice treated with 4-MeH compared to those treated with the vehicle (Figure 1). These results strongly suggest that 4-MeH exacerbates the progression of EAE in mice.

### 2.2. Effects of the H4R Agonist on NF-κB p65 Expression in EAE Mice

**Figure 2:**
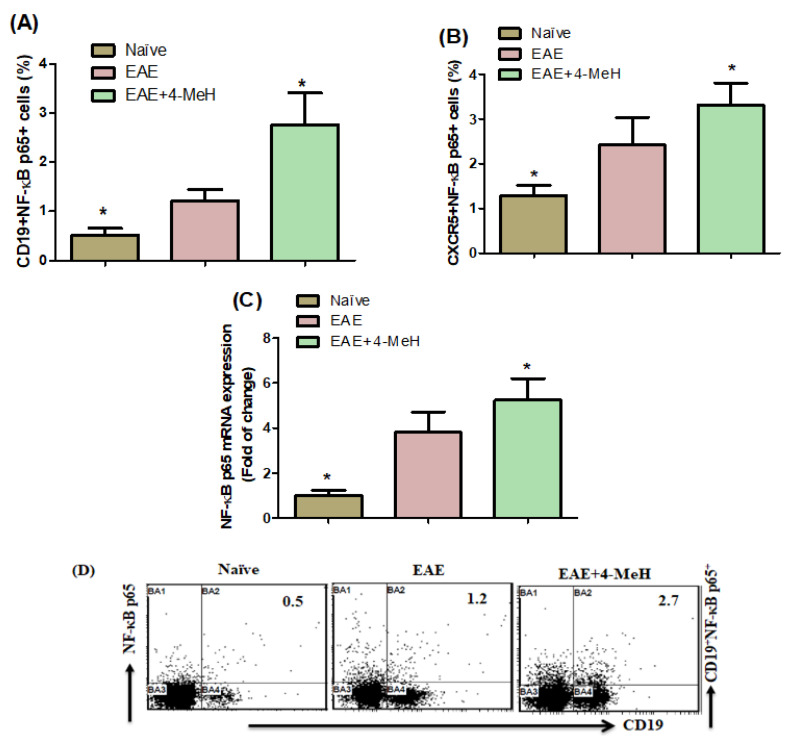
Flow cytometric analysis indicates the effects of 4-MeH on NF-κB p65- expressing (**A**) CD19^+^ and (**B**) CXCR5^+^ cells in the spleen. (**C**) shows the NF-κB p65 mRNA expression in the brain. SJL mice were immunized with PLP139-151 to induce EAE. Then, the mice were intraperitoneally treated with 4-MeH (30 mg/kg) or vehicle daily from day 14 to day 42, and the brain tissues were isolated. The NF-κB p65 mRNA expression was estimated using RT-PCR. (**D**) Representative fluorescence-activated cell sorter dot plots of CD19^+^NF-κB p65^+^ cells are shown. All data are shown as mean ± SD (n = 6). The level of significance was set at * *p* < 0.05.

Subsequently, we examined the impact of the H4 receptor (H4R) agonist on CD19^+^ and CXCR5^+^ cells expressing NF-κB p65 in the spleens of EAE mice treated with Normal Controls (NC), vehicle, and 4-MeH. The proportion of CD19^+^NF-κB p65^+^ and CXCR5^+^NF-κB p65^+^ cells showed a notable increase in 4-MeH-treated EAE mice compared with those treated with the vehicle (Figure 2A,B). To further elucidate the mechanism by which H4R agonists operate in EAE mice, we conducted an RT-PCR analysis to assess changes in the mRNA expression of NF-κB p65 in the brain. The findings indicated significant upregulation of NF-κB p65 mRNA expression in the 4-MeH-treated EAE mice compared with the vehicle-treated EAE mice (Figure 2C). These outcomes imply that the administration of 4-MeH substantially impacted NF-κB expression in the spleen and brain of EAE mice.

### 2.3. Effects of H4R Agonist on GM-CSF Expression in EAE Mice

**Figure 3:**
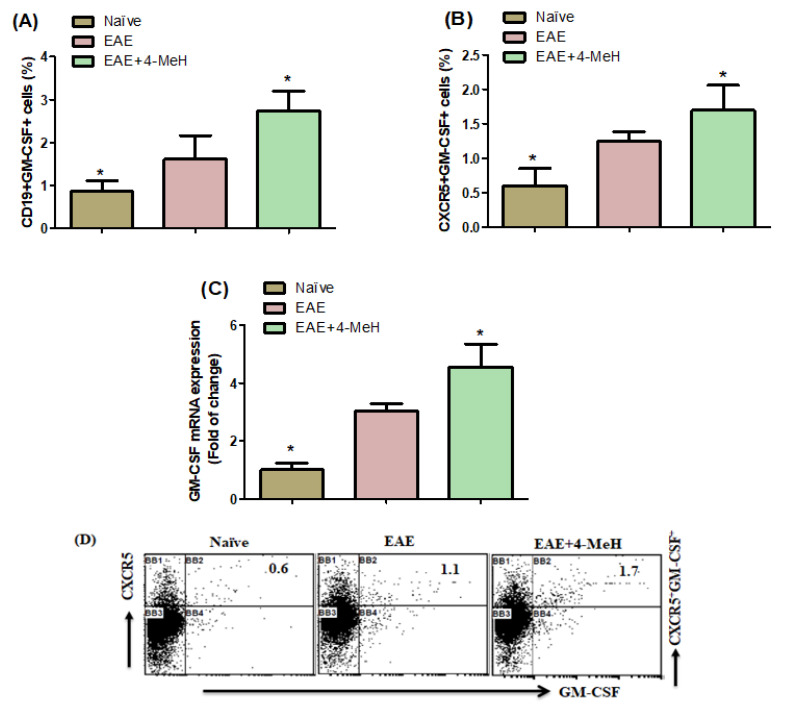
Flow cytometric analysis indicates the effects of 4-MeH on GM-CSF-expressing (**A**) CD19^+^ and (**B**) CXCR5^+^ cells in the spleen. (**C**) GM-CSF mRNA expression in the brain using RT-PCR was shown. SJL mice were immunized with PLP139-151 to induce EAE. Then, the mice were intraperitoneally treated with 4-MeH (30 mg/kg) or vehicle daily from day 14 to day 42, and the brain tissues were isolated. The GM-CSF mRNA expression was estimated using RT-PCR (**D**) Representative fluorescence-activated cell sorter dot plots of CXCR5^+^GM-CSF^+^ cells. All data are shown as mean ± SD (n = 6). The level of significance was set at * *p* < 0.05.

Examining the impact of H4 Receptor (H4R) agonists on CD19^+^ and CXCR5^+^ cells expressing GM-CSF, we observed a noteworthy increase in the population of GM-CSF-expressing CD19^+^ and CXCR5^+^ cells in EAE mice treated with 4-MeH compared with those treated with the vehicle (Figure 3A,B). Additionally, the mRNA expression level of GM-CSF in the brains of 4-MeH-treated EAE mice was upregulated compared with that in vehicle-treated EAE mice (Figure 3C). These results show that 4-MeH exaggerates MS progression by upregulating GM-CSF expression in EAE.

### 2.4. Effects of H4R Agonist on MCP-1 Expression in EAE Mice

**Figure 4:**
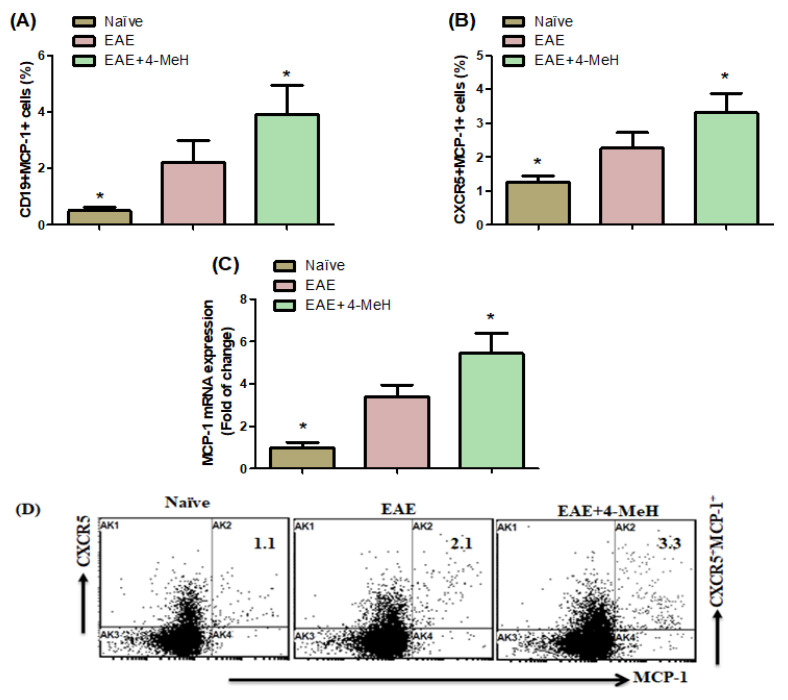
(**A**,**B**) Flow cytometric analysis indicates the effects of 4-MeH on MCP-1-expressing (**A**) CD19^+^ and (**B**) CXCR5^+^ cells in the spleen. (**C**) MCP-1 mRNA expression in the brain using RT-PCR is shown. SJL mice were immunized with PLP139-151 to induce EAE. Then, the mice were intraperitoneally treated with 4-MeH (30 mg/kg) or vehicle daily from day 14 to day 42, and the brain tissues were isolated. The MCP-1 mRNA expression was estimated using RT-PCR. (**D**) Representative fluorescence-activated cell sorter dot plots of CXCR5^+^MCP-1 cells. All data are shown as mean ± SD (n = 6). The level of significance was set at * *p* < 0.05.

Administration of 4-MeH resulted in an elevation of CD19^+^MCP-1^+^ and CXCR5^+^MCP-1^+^ cells and an increased percentage of these cell populations in EAE mice compared with the vehicle-treated group (Figure 4A,B). Moreover, the mRNA expression level of MCP-1 in the brains of EAE mice was augmented following treatment with 4-MeH compared with the vehicle-treated EAE mice (Figure 4C). These findings provide evidence that H4R agonist in EAE mice also regulates MCP-1. These results suggested that MVC treatment exerts a therapeutic effect during RA development.

### 2.5. Effects of H4R Agonist on TNF-α Expression in EAE Mice

**Figure 5:**
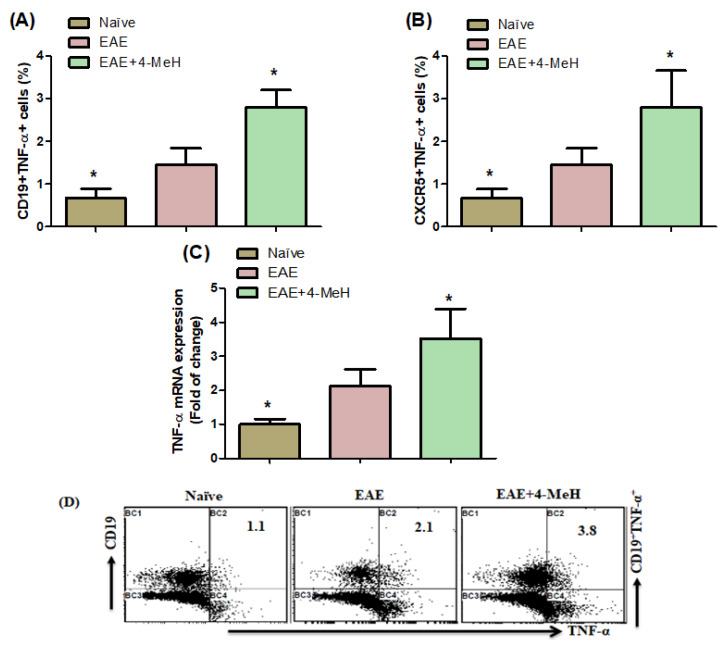
(**A**,**B**) Flow cytometric analysis indicates the effects of 4-MeH on TNF-α-expressing (**A**) CD19^+^ and (**B**) CXCR5^+^ cells in the spleen. (**C**) TNF-α mRNA expression in the brain is shown. SJL mice were immunized with PLP139-151 to induce EAE. Then, the mice were intraperitoneally treated with 4-MeH (30 mg/kg) or vehicle daily from day 14 to day 42, and the brain tissues were isolated. The TNF-α mRNA expression was estimated using RT-PCR. (**D**) Representative fluorescence-activated cell sorter dot plots of CD19^+^TNF-α^+^ cells. All data are shown as mean ± SD (n = 6). The level of significance was set at * *p* < 0.05.

To evaluate whether H4R agonists modulate the expression of pro-inflammatory cytokines, we examined them in EAE mice. Thus, we measured the expression of TNF-α in EAE mice. Our findings showed that EAE mice treated with 4-MeH had a higher percentage of CD19^+^TNF-α^+^ and CXCR5^+^TNF-α^+^ cells than vehicle-treated EAE mice (Figure 5A,B). The TNF-α mRNA expression was also higher in 4-MeH-treated EAE mice than in those treated with a vehicle (Figure 5C). Based on these results, it can be suggested that H4R agonists could be involved in the molecular mechanisms implicated in MS.

### 2.6. Effects of H4R Agonist on IL-6 Expression in EAE Mice

**Figure 6:**
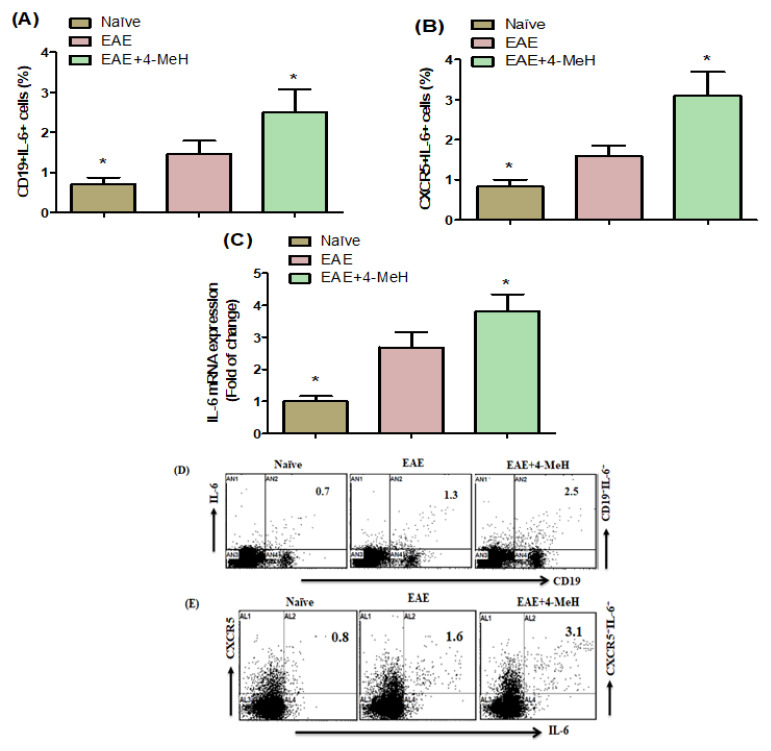
Flow cytometric analysis indicates the effects of 4-MeH on IL-6-expressing (**A**) CD19^+^ and (**B**) CXCR5^+^ cells in the spleen. (**C**) IL-6 mRNA expression was analyzed in the brain using RT-PCR. SJL mice were immunized with PLP139-151 to induce EAE. Then, the mice were intraperitoneally treated with 4-MeH (30 mg/kg) or vehicle daily from day 14 to day 42, and the brain tissues were isolated. The IL-6 mRNA expression was estimated using RT-PCR. (**D**,**E**) Representative fluorescence-activated cell sorter dot plots of CD19^+^IL-6^+^ and CXCR5^+^IL-6^+^ cells as shown. All data are shown as mean ± SD (n = 6). The level of significance was set at * *p* < 0.05.

Figure 6A,B illustrate that in EAE mice, 4-MeH produced a significant increase in CD19^+^IL-6^+^ and CXCR5^+^IL-6^+^ cells compared with the vehicle. In the brain of 4-MeH-treated EAE mice, there was a notable increase in the amount of IL-6 mRNA expression compared to vehicle-treated EAE mice (as shown in Figure 6C). These findings conclude that 4-MeH plays a significant part in the upregulation of IL-6 in EAE mice.

## 3. Discussion

The CNS myelin protein, myelin proteolipid protein (PLP), is significant. Epitopes of PLP capable of inducing EAE in multiple strains of mice have been identified [48]. In SJL (H-2s) mice, two encephalitogenic epitopes of PLP, PLP-139–151 and PLP-178–191, are the major ones [49,50]. These two epitopes bind with a binding affinity of K50 < 1 μM to I-As [48]), but PLP-139–151 is unusually dominant. MS, a chronic autoimmune inflammatory disease affecting the CNS and resulting in axonal damage and neuronal demyelination [51], is believed to be initiated by pathogenic CD4^+^ T cells that produce inflammatory cytokines [52]. Nonetheless, research over the past decade has shown that B cells play a critical role in MS pathogenesis, in addition to other regulatory functions [53,54]. B cells are involved in antigen presentation, so they could have a possible inflammatory role in the CNS [55]. Based on our hypothesis that H4R agonists are linked to MS development and progression, our study indicates that 4-MeH can compound clinical signs in EAE mice and increase EAE pathogenesis, thereby supporting our hypothesis. This is the first time this has been demonstrated.

Various master transcription factors regulate inflammatory cell activity, and NF-κB is one of them. During disease progression, NF-κB is crucial in the resident cells of the CNS [56]. Various studies suggest that neuroinflammation caused by NF-κB contributes to neuronal degeneration in the EAE model of MS [57]. In the pathogenesis of autoimmune demyelinating diseases, the NF-κB signaling cascade is vital for producing EAE pathology [58]. Additionally, microglia activation development is associated with NF-κB signaling [59]. The present study demonstrated that the 4-MeH-treated EAE mice had more CD19^+^NF-κB^+^ and CXCR5^+^NF-κB^+^ cells than vehicle-treated mice. The experimental results indicate that NF-κB signaling is influenced by H4R agonists in EAE-diseased mice, as shown by the upregulation of the NF-κB mRNA expression in 4-MeH-treated EAE mice. Primarily at disease onset, the H4R agonist plays a crucial role in NF-κB signaling in the EAE model. These results suggest the possible involvement of the H4R agonist in the development of MS. These results underline the need to determine further the relevance of the H4R agonist in MS disease severity.

Individuals with relapsing-remitting MS have been found to exhibit increased levels of GM-CSF in their cerebrospinal fluid, as noted in reference [60]. Furthermore, mice that lack GM-CSF were resistant to EAE induction [61]. Recent data have also shown that GM-CSF is activated in the disease progression and pathogenicity of EAE [19]. In contrast, Mice that have the GM-CSF gene knocked out and T cells without GM-CSF cannot induce EAE or migrate to the CNS, as noted in reference [62]. Additionally, an increased occurrence of GM-CSF-producing Th cells has been observed in patients with MS compared to those suffering from other neurological disorders [63]. Patients with MS have a greater number of GM-CSF-producing B cells. GM-CSF performs an autocrine function in B cell survival and can excite myeloid cells [64], which may impact MS. The study demonstrated that MS exacerbation was amplified by 4-MeH, which increased the number of GM-CSF-expressing CD19^+^ and CXCR5^+^ cells and stimulated GM-CSF mRNA. Our findings suggest that the H4R agonist dysregulates pro-inflammatory responses in EAE mice and could be significantly involved in the initiation and progression of MS.

Chemokines play key roles in inflammatory changes contributing to MS. MCP-1 is essential in many neurodegenerative brain diseases [65]. Increased MCP-1 production has been observed in microglia and fetal astrocytes [66]. Moreover, research has identified the presence of MCP-1 expression in neurons located in the cerebral cortex, hippocampal formation, brainstem, and cerebellum, as referenced in [67]. Additionally, a study from the past found a correlation between elevated MCP-1 expression and relapse frequency and clinical severity in individuals with relapsing EAE [68]. The present study demonstrated that 4-MeH upregulated Mcp1 expression in EAE mice, providing evidence for the stimulatory effect of H4R agonist in MCP-1 presentation in an EAE model. These results indicated that the upregulation of MCP-1 could be associated with the immune dysfunction observed in MS. Our study reveals a critical role for H4R agonists in the induction of EAE. Accordingly, H4R agonists might provide an aggregative approach to understanding the mechanisms underlying MS and assist in developing therapeutics against MS.

NF-κB is a transcription factor that triggers the expression of inflammation-related genes such as TNF-α and IL-6. TNF-α is a potent activator of this factor, as mentioned in reference [69]. Multiple studies have demonstrated the involvement of TNF-α in the progression of EAE, with TNF-α expression levels shown to increase during relapses of MS, suggesting a significant effect on the pathogenesis and progression of the disease [70]. Elevated levels of TNF-α have been detected in the CNS during acute episodes of EAE and in patients with MS [28,71], signifying its connection with disease advancement [72]. In this study, it was observed that treatment with the H4R agonist 4-MeH resulted in increased CD19^+^TNF-α^+^ and CXCR5^+^TNF-α^+^ cells in EAE mice, and the treatment also stimulated the expression of Tnfα mRNA in their brain. These outcomes suggest that the H4R plays an important role in increasing inflammatory mediators and is connected with disease severity in individuals with MS. This implies that H4R functions as a pro-inflammatory mediator in the development of EAE.

Studies indicate that mice without IL-6 are not susceptible to EAE [73], and individuals with MS present an augmented expression of IL-6 [74]. Patients diagnosed with neuromyelitis optica, primary progressive MS, and relapsing-remitting MS have also shown elevated IL-6 levels [75]. Furthermore, increased production of IL-6 was observed in monocytes extracted from individuals with MS [29]. After administering 4-MeH treatment to EAE mice, we observed an increase in CD19^+^ and CXCR5^+^ B cells expressing IL-6, corresponding to Il6 mRNA expression in the mice’s brains. These results imply that H4R agonists can significantly stimulate pro-inflammatory cytokines. Therefore, we inferred that the H4R agonist elevates the expression of pro-inflammatory cytokines, contributing to its aggregative development through the upregulation of IL-6 in MS. Further investigation is necessary to clarify the exact influence of the H4R agonist in MS.

## 4. Material and Methods

### 4.1. Reagents

Axon MedChem, located in Groningen, Netherlands, was the source of the 4-MeH compound. BioLegend, based in San Diego, CA, USA, provided the anti-CD19 monoclonal antibodies, anti-CXCR5 monoclonal antibodies, anti-NF-κB p65 FITC, anti-GM-CSF FITC, MCP-1 APC, anti-IL-6 PE/Dazzle, anti-TNF-α PE anti-mouse monoclonal antibodies, as well as RBC lysis, permeabilization buffers, and fixation/permeabilization buffers. Sigma-Aldrich, based in St. Louis, MO, USA, provided Phorbol 12-myristate 13-acetate, ionomycin, and RPMI 1640 medium. Golgi plugs were provided by BD Biosciences, San Jose, CA, USA. GenScript, based in Piscataway, NJ, USA, supplied the primers required to evaluate gene expression. The cDNA Reverse Transcription kit and SYBR^®^ Green PCR Master Mix were provided by Applied Biosystems, Paisley, UK, while the TRIzol reagent was obtained from Invitrogen, Carlsbad, CA, USA.

### 4.2. Experimental Animals

Jackson Laboratories, located in Bar Harbor, ME, USA, was the source of female Swiss Jim Lambert (SJL) mice. The mice, weighing 20–25 g and aged 8–10 weeks, were kept in secure, pathogen-free environments and given standard rodent food with unlimited access to water at King Saud University’s animal facility. The research team made every effort to ensure that the animals did not undergo unnecessary discomfort. Research procedures on the animals followed approved methods under the oversight of the Institutional Animal Care and Use Committee. The protocol for the animal study received approval from the College of Pharmacy’s Ethical Committee at King Saud University, with an Ethical Reference Number of KSU-SE-21-50.

### 4.3. EAE Induction and Administration

To induce EAE, mice were given subcutaneous injections of PLP139–151 peptide at four sites, with 200 µg emulsified with complete Freund’s adjuvant at 50 μL per site, following methods discussed in previous reports [76,77,78]. On immunization day, the mice received intraperitoneal injections of 200 ng pertussis toxin from Hooke Laboratories, USA, following the methods described in prior studies [76,77,78]. The mice were then randomly assigned to one of three groups: the naive group, which received saline (n = 6), the immunized group with a vehicle (EAE; n = 6); and the immunized group with the H4R agonist 4-MeH (EAE+4-MeH; n = 6). To investigate the effect of 4-MeH administration on the EAE mouse model, 4-MeH was administered i.p. at 30 mg/kg from day 14 to day 42. The dose of 4-MeH was selected based on previous studies [79,80].

### 4.4. EAE Clinical Scoring

The researchers clinically assessed EAE daily, rating its severity on a scale of 0–5, where 0 indicates no observable symptoms, 1 indicates tail paralysis, 2 indicates partial hind limb paralysis, 3 indicates complete hind limb paralysis, 4 indicates paralysis in both fore and hind limbs, and 5 indicates being moribund or dead [77,78,81].

### 4.5. Flow Cytometric Analysis

Conjugated antibodies to CD19, CXCR5, NF-κB p65, GM-CSF, MCP-1, IL-6, and TNF-α were used to analyze flow cytometry. For spleen cell staining, the following conjugated antibodies were utilized: anti-CD19, anti-CD19 FITC, anti-CD19 APC, anti-CXCR5 PE/Dazzle, anti-CXCR5 APC CY7, anti-NF-κB p65 FITC, anti-GM-CSF FITC, MCP-1 APC, anti-IL-6 PE/Dazzle, and anti-TNF-α PE- by procedures discussed in previous research [82,83]. Before staining, splenocytes were incubated with PMA/ionomycin and Golgi-Plug for four hours. Surface staining for CD19 and CXCR5 was subsequently performed on washed cells, followed by fixation and permeabilization, along with staining for NF-κB p65, GM-CSF, MCP-1, IL-6, and TNF-α using fluorescent antibodies. The Beckman Coulter FC500 flow cytometer acquired ten thousand cell events, and the data were assessed using CXP software from Beckman Coulter, Indianapolis, IN, USA.

### 4.6. Real-Time PCR

The TRIzol reagent from Life Technologies, Paisley, UK, was used to isolate total RNA from the brain. The quality and integrity of the RNA were then verified with a NanoDrop 2000c spectrophotometer from Thermo Fisher Scientific. A high-capacity cDNA reverse transcription kit from Applied Biosystems in Foster City, USA, was used to prepare cDNA, followed by real-time PCR with an SYBR^®^ Green PCR master mix from Applied Biosystems, as discussed in previous studies [82,83]. RT-PCR assays were conducted using the following primers from Genscript, Piscataway, USA. NF-κB p65, F:5′-TGCAGAGAGACTGATCGGGA-3′ and R:5′-GCCTGGTCCCGTGAAATACA-3′; GM-CSF, F:5′-AGCTTTACGAGAGCTCTTTTGC-3′ and R:5′-CACATCCTCCTCAGGACCTT-3′; MCP-1, F:5′-CAAAGCCAGGGGCCTTTTTC-3′ and R:5′-TACCAGGAGCCAGGCATAGT-3′; IL-6, F:5′-GCCTTCTTGGGACTGATGCT-3′ and R:5′- GACAGGTCTGTTGGGAGTGG-3′; TNF-α, F:5ʹ-GGACTAGCCAGGAGGGAGAA -3′ and R:5′-CGCGGATCATGCTTTCTGTG-3′; GAPDH, F:5′-TGTGAACGGATTTGGCCGTA-3′ and R:5′-ACTGTGCCGTTGAATTTGCC-3′. Real-time PCR was performed using an SYBR^®^ Green PCR master mix (Applied Biosystems). Relative changes in gene expression were determined using the 2^−ΔΔCT^ method [84] with GAPDH as the reference gene.

### 4.7. Statistical Analysis

GraphPad PRISM 5.0 software from GraphPad Software in San Diego, CA, USA, was used to conduct statistical analyses. The mean and standard deviation are presented in the data. One-way ANOVA was conducted, followed by Bonferroni’s post hoc comparison test for statistical analysis. The level of statistical significance was set at *p* < 0.05, indicating that the results were statistically significant.

## 5. Conclusions

The current study demonstrated for the first time that 4-MeH administration significantly deteriorated clinical symptoms in EAE mice. 4-MeH likely has an aggregative effect by upregulating pro-inflammatory mediators. Our findings indicate that H4R agonists contribute considerably to the onset and advancement of MS by increasing the expression of pro-inflammatory mediators. In conclusion, H4R agonists are a crucial component of the pathology of MS and EAE experimental models, and additional research is required to elucidate the molecular mechanisms underlying the neuroinflammatory effects of H4R agonists.

## Data Availability

All data presented in this study are available on reasonable request from the corresponding author.

## Abbreviations

4-methylhistamine (4-MeH); Histamine H4 receptor (H4R); experimental autoimmune encephalomyelitis (EAE); multiple sclerosis (MS); Swiss Jim Lambert (SJL); Microliter (μL); CXC motif chemokine receptor 5 (CXCR5); central nervous system (CNS); Cluster of differentiation (CD); Phorbol 12-myristate 13-acetate (PMA); Nuclear factor kappa-light-chain-enhancer of activated B cells (NF-κB); Proteolipid protein (PLP); Granulocyte-macrophage colony-stimulating factor (GM-CSF); Tumor necrosis factor alpha (TNF-α); Interleukin 6 (IL-6); Monocyte Chemoattractant Protein-1 (MCP-1); Intraperitoneally (i.p.); Phycoerythrin (PE); Fluoroisothiocyanate (FITC); allophycocyanin (APC); Messenger RNA (mRNA); Reverse transcription polymerase chain reaction (RT-PCR); Complementary DNA (cDNA); Glyceraldehyde 3-phosphate dehydrogenase (GAPDH); Roswell Park Memorial Institute (RPMI) 1640
